# More home births during the COVID‐19 pandemic in the Netherlands

**DOI:** 10.1111/birt.12646

**Published:** 2022-05-12

**Authors:** Corine J. M. Verhoeven, José Boer, Marjolein Kok, Marianne Nieuwenhuijze, Ank de Jonge, Lilian L. Peters

**Affiliations:** ^1^ Department of Midwifery Science, AVAG/Amsterdam Reproduction and Development Center Amsterdam University Medical Centres, Vrije Universiteit Amsterdam Amsterdam Netherlands; ^2^ Department of Midwifery, School of Health Sciences University of Nottingham Nottingham UK; ^3^ Department of Obstetrics and Gynaecology Maxima Medical Centre Veldhoven the Netherlands; ^4^ Department of Obstetrics and Gynaecology, Amsterdam Reproduction and Development Center Amsterdam University Medical Centre, Universiteit van Amsterdam Amsterdam the Netherlands; ^5^ Research Centre for Midwifery Science Zuyd University Maastricht the Netherlands; ^6^ CAPHRI School for Public Health and Primary Care Maastricht University Maastricht the Netherlands; ^7^ Department of Midwifery Science, AVAG/Amsterdam Public Health Research Institute Amsterdam University Medical Centres, Vrije Universiteit Amsterdam Amsterdam Netherlands; ^8^ Department of General Practice and Elderly Care Medicine University of Groningen, University Medical Centre Groningen Groningen the Netherlands

**Keywords:** COVID, low‐risk pregnant women, mode of birth, place of birth, pregnancy and birth

## Abstract

**Background:**

The aim of this observational study was to examine whether the course of pregnancy and birth and accompanying outcomes among low‐risk pregnant women changed in the COVID‐19 pandemic compared to the prepandemic period.

**Methods:**

We analyzed data from the Dutch Midwifery Case Registration System (VeCaS). Differences in the course of pregnancy and birth, and accompanying maternal and neonatal outcomes, were calculated between women pregnant during the initial months of the COVID‐19 pandemic (March 1 to August 3, 2020) and the prepandemic period (March 1–August 3, 2019). We also conducted a stratified analysis by parity.

**Results:**

We included 5913 low‐risk pregnant women of whom 2963 (50.1%) were pregnant during the first surge of the COVID‐19 pandemic, and 2950 (49.9%) in the prepandemic period. During the COVID‐19 pandemic, more women desired and had a home birth. More women used pain medication and fewer had an episiotomy in the COVID‐19 period than prior. Multiparous women had a higher suspected rate of fetal growth restriction during COVID; however, the actual rate of small for gestational age infants was not significantly increased. We observed no differences for onset and augmentation of labor or for mode of birth, though the rate of vaginal births increased.

**Conclusions:**

During the COVID‐19 pandemic, there was a higher rate of planned and actual home birth, and suspected growth restriction and a lower rate of episiotomy among low‐risk pregnant women in the Netherlands.

## 1 INTRODUCTION

Since 2020, the world has been dealing with the coronavirus disease 2019 (COVID‐19) pandemic. The first case of COVID‐19 appeared in Wuhan, China in December 2019,^1^ leading to an outbreak in the beginning of January 2020. By February 2020, SARS‐CoV‐2 was globally disseminated and, due to the increase in COVID‐19 cases, more and more countries entered into partial or nation‐wide lock downs. Globally, as of April 12, 2022, there have been 497.960.492 confirmed cases of COVID‐19, including 6.181.850 deaths, reported to WHO.^2^

The pandemic put high pressure on the health system and urgent adjustments were made.^3^, ^4^ Globally, as well as in the Netherlands, safety measures were taken to protect both patients and staff from infection. Routine medical care was scaled down to the minimum care necessary, and measures were taken to slow down transmission.^3^, ^4^ These actions had significant impacts for maternity care organizations around the world. In maternity care, face‐to‐face consultations were diminished, and remote consultations were made possible via various methods. During the first wave of the pandemic, many birth settings did allow the presence of the woman’s partner, but no other birth companion during labor.

The Dutch Royal College for Midwives (KNOV) and the Dutch Society of Obstetrics and Gynaecology (NVOG) provided a guideline with recommendations to rationalize care and minimize face‐to‐face interaction with health professionals during the pandemic.^5^, ^6^ Prenatal care was reduced from 13 to 7 consultations for term pregnancies, and women were asked to come to appointments alone. Before a face‐to‐face consultation, pregnant women received a phone call asking whether they had any COVID‐19‐related symptoms and to discuss their health issues. This was done to keep the duration of the face‐to‐face contacts to a minimum. Two routine prenatal ultrasounds continued to be offered. Most hospitals instituted a limit of one adult visitor for each woman in labor and delivery units, and visitors other than the woman’s partner were not permitted to postpartum units. The measures taken impacted the organization and utilization of maternity care profoundly.^5^, ^6^

In the Netherlands, low‐risk pregnant women are cared for by an independent primary care midwife and have the choice to give birth at home, in a birth center or in a hospital with midwife‐led care. If problems during pregnancy or birth arise, women are referred to hospital‐based, obstetrician‐led care.

Little is known about the care of low‐risk women under the care of primary care midwives during pandemic situations. To date, limited data on the perinatal care of these women are available. For example, the effect of the COVID‐19 pandemic on women’s decision on the place of birth is not known, though one study from the United States recently identified a 23% increase in home births between 2019 and 2020.^7^ In addition to changes in health care access, the pandemic and societal restrictions led to changes in lifestyle, as well as in physical and mental health. These too may have impacted maternal and perinatal outcomes. Therefore, the aim of this study was to examine whether the course of pregnancy and birth of low‐risk women who started their care in primary midwifery care, and the accompanying maternal and neonatal health outcomes differed during the first wave of the COVID‐19 pandemic (2020) compared to the prepandemic period (2019).

## 2 METHODS

### 2.1 Study design

Data were analyzed from the Midwifery Case Registration System (Verloskundig Casusregistratie Systeem, VeCaS).^8^ This system includes routinely collected data from electronic primary midwifery care registration systems (i.e., Orfeus and Vrumun) used by 39 Dutch Midwifery care practices across the Netherlands. The VeCaS database contains data on a population that is comparable to the national population in primary midwife‐led care in the Netherlands.^8^ For the present study, we analyzed data on women who gave birth in the COVID‐pandemic period from March 2020 to August 2020 and in the prepandemic period (March–August 2019). All women in the database provided informed consent for the use of their anonymized data.

### 2.2 Participants

Participants were selected from the VeCaS database that consists of low‐risk pregnant women in the care of primary care midwives. It includes healthy women, with a singleton pregnancy who are eligible for a midwife‐led birth at the start of their pregnancy. For this study, we selected women with singleton pregnancies, known parity, who gave birth from 24 weeks of gestation onward, and had at least one appointment with a primary care midwife after 24 weeks of gestation.

### 2.3 Outcomes

We extracted data on maternal characteristics, pregnancy and birth characteristics, and maternal and neonatal outcomes. Maternal demographic characteristics included maternal age, marital status (single, in a relationship), migration background (Dutch, Western non‐Dutch, non‐Western, other), smoking status during pregnancy (no, stopped smoking in first trimester, or yes), prepregnancy body mass index (BMI), maternal socioeconomic status (national percentiles based on employment, education and income level of the residential postal code area categorized into below the 25th percentile (low), 25th to 75th percentile (medium), and above the 75th percentile (high)),^9^ and urbanization grade. The urbanization grade was based on residential postal codes, that is, the mean number of addresses per square kilometer.^10^ For this study, we used the following categories: (a) extremely urbanized (≥2500 addresses/km^2^), (b) strongly urbanized (1500–2500 addresses/km^2^), (c) moderately urbanized (1000–1500 addresses/km^2^), (d) hardly urbanized (500–1000 addresses/km^2^), and (e) not urbanized (<500 addresses/km^2^).^9^

Pregnancy characteristics included parity and pregnancy complications (hypertensive disorders, gestational diabetes mellitus, and suspected fetal growth restriction, assessed by ultrasound, and placenta previa).^11^, ^12^ Hypertensive disorder was operationalized as one blood pressure measurement after 20 weeks’ gestation with a diastolic pressure ≥90 and/or a systolic pressure ≥140 mmHg.^13^ The following birth characteristics were collected: level of care at the onset of labor (primary midwifery‐led or secondary obstetrician‐led care), onset of labor (e.g., spontaneous, induction), augmentation of labor, pain medication (e.g., pethidine, remifentanil, epidural), preferred (as discussed during pregnancy) and actual place of birth (e.g., home, hospital), and mode of birth (e.g., vaginal birth, cesarean birth). Maternal health outcomes were the amount of blood loss after the birth, 3rd/4th degree tears, and episiotomy. Neonatal outcomes were gestational age at birth, that is, extremely preterm (<28 weeks), very preterm (28–31 + 6), moderate to late preterm (32–36 + 6), term (37–41 + 6), and post‐term (≥42 weeks).^14^ Next, data on gender, birthweight in percentiles according to the Dutch Perined birthweight charts,^15^, ^16^ Apgar score after 5 min, and perinatal mortality <28 days after birth were extracted.

### 2.4 Statistical analysis

Descriptive statistics were used to report the prevalence of maternal, pregnancy and birth characteristics, maternal and neonatal health outcomes for the total population, as well as for nulliparous and multiparous women in the prepandemic period (2019) and COVID‐19 pandemic period (2020). Prevalences were reported with numbers and valid percentages. To assess differences in characteristics and outcomes among women who were pregnant in the COVID‐19 pandemic (2020) compared to those who were pregnant in the prepandemic period (2019), we calculated chi‐squared tests for the total population, as well as for nulliparous and multiparous women.

All data were analyzed in SPSS version 26.0 (SPSS Inc., Chicago, IL, USA). A *P*‐value below 0.05 was considered statistically significant.

## 3 RESULTS

**FIGURE 1:**
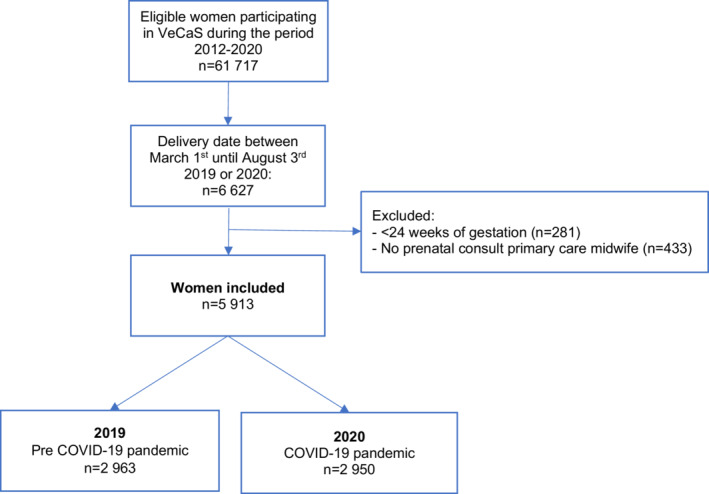
Flowchart of the study population

We extracted data for 6627 women who gave birth to singletons during the first surge of the COVID‐19 pandemic (2020) or in the same period in the previous year (2019). We excluded 714 (11%) women for the following reasons: pregnancy ended before 24 weeks gestation (n = 281) and women who had no bookings scheduled with their primary care midwives after 24 weeks gestation (n = 433). In total, we included 5913 women, 2963 (50.1%) of them were pregnant in the COVID‐19 pandemic, whereas 2950 (49.9%) were pregnant in the prepandemic period (Figure 1).

Women who were pregnant during the COVID‐19 pandemic (2020) did not differ on maternal characteristics, compared with women who were pregnant in the prepandemic period (2019). The mean maternal age was 31.4 (SD: 4.4) and 31.3 (SD: 4.4) years, respectively. Most of the women included had a partner/spouse and were Dutch. A quarter of them had a low socioeconomic status and one third lived in an extremely/strongly urbanized environment (Table 1).

**TABLE 1:** Maternal characteristics of the included VeCaS‐population of low‐risk pregnant women in the prepandemic (2019, n = 2963) and COVID‐19 pandemic period (2020, n = 2950), total n = 5913

	2019			2020			Statistical differences between 2019–2020		
	Total	Primiparous	Multiparous	Total	Primiparous	Multiparous	Total	Primiparous	Multiparous
	n = 2963	n = 1317	n = 1646	n = 2950	n = 1319	n = 1631			
	100%	44%	56%	100%	45%	55%			
	n (%)	n (%)	n (%)	n (%)	n (%)	n (%)	*P*‐value	*P*‐value	*P*‐value
Maternal age (years)									
≤25	288 (9.7)	210 (15.9)	78 (4.7)	267 (9.1)	191 (14.5)	76 (4.7)	0.467	0.239	0.965
26–30	712 (24.0	434 (33.0)	278 (16.9)	674 (22.8)	403 (30.6)	271 (16.6)			
31–35	1306 (44.1	517 (39.3)	789 (47.9)	1323 (44.8)	549 (41.6)	774 (47.5)			
≥35	657 (22.2)	156 (11.8)	501 (30.4	686 (23.3)	176 (13.3)	510 (31.3)			
Marital status									
Partner/spouse	2865 (98.0)	1265 (98.0)	1600 (98.0)	2850 (98.1)	1269 (97.6)	1581 (98.4)	0.803	0.520	0.319
Single	59 (2.0)	26 (2.0)	33 (2.0)	56 (1.9)	31 (2.4)	25 (1.6)			
Missing	39	26	13	44	19	25			
Socioeconomic status									
Low (<p25)	788 (26.8)	371 (28.3)	417 (25.6)	750 (25.6)	333 (25.4)	417 (25.8)	0.414	0.247	0.428
Medium (p25‐p75)	1602 (54.4)	698 (53.2)	904 (55.4)	1596 (54.5)	729 (55.6)	867 (53.6)			
High (>p75)	554 (18.8)	244 (18.6)	310 (19.0)	585 (20.0)	250 (19.1)	335 (20.7)			
Missing	19	4	15	19	7	12			
Level of urbanization^a^									
Extremely urbanized	358 (12.1)	192 (14.6)	166 (10.1)	328 (11.1)	169 (12.8)	159 (9.8)	0.199	0.335	0.671
Strongly urbanized	618 (20.9)	289 (22.0)	329 (20.1)	629 (21.4)	301 (22.9)	328 (20.2)			
Moderately urbanized	701 (23.7)	302 (22.9)	399 (24.4)	645 (21.9)	274 (20.8)	371 (22.8)			
Hardly urbanized	745 (25.2)	312 (23.7)	433 (26.4)	804 (27.3)	339 (25.7)	465 (28.6)			
Not urbanized	532 (18.0)	221 (16.8)	311 (19.0)	538 (18.3)	234 (17.8)	304 (18.7)			
Missing	9	1	8	6	2	4			
Migration Background^a^									
Dutch	2312 (80.3)	1032 (81.1)	1280 (79.6)	2288 (79.0)	1033 (80.3)	1255 (77.9)	0.074	0.286	0.065
Western Non‐Dutch	208 (7.2)	101 (7.9)	107 (6.7)	252 (8.7)	105 (8.2)	147 (9.1)			
Non‐Western	348 (12.1)	136 (10.7)	212 (13.2)	336 (11.6)	138 (10.7)	198 (12.3)			
Other	12 (0.4)	3 (0.2)	9 (0.6)	21 (0.7)	10 (0.8)	11 (0.7)			
Missing	83	45	38	53	33	20			
Smoking during pregnancy									
No	2551 (88.5)	1120 (87.0)	1431 (89.7)	2530 (88.0)	1102 (85.6)	1428 (89.9)	0.371	0.159	0.969
Stopped in first trimester	181 (6.3)	98 (7.6)	83 (5.2)	205 (7.1)	124 (9.6)	81 (5.1)			
Yes	152 (5.3)	70 (5.4)	82 (5.1)	141 (4.9)	62 (4.8)	79 (5.0)			
Missing	79	29	50	74	31	43			
Prepregnancy body mass index									
Underweight (<18.5)	76 (2.6)	38 (2.9)	38 (2.4)	74 (2.5)	39 (3.0)	35 (2.2)	0.982	0.226	0.468
Normal weight (18.5–24.9)	1783 (60.9)	848 (64.7)	935 (57.8)	1766 (60.5)	818 (62.3)	948 (59.0)			
Pre‐obesity (25–29.9)	712 (24.3)	280 (21.4)	432 (26.7)	725 (24.8)	312 (23.8)	413 (25.7)			
Obesity (>30)	356 (12.2)	144 (11.0)	212 (13.1)	354 (12.2)	144 (10.9)	210 (13.1)			
Missing	36	7	29	31	6	25			

a Level of urbanization was categorized as: (a) extremely urbanized (≥2500 addresses/km^2^), (b) strongly urbanized (1500–2500 addresses/km^2^), (c) moderately urbanized (1000–1500 addresses/km^2^), (d) hardly urbanized (500–1000 addresses/km^2^), and (5) not urbanized (<500 addresses/km^2^).

b For migration background, the definitions of Statistics Netherlands (Centraal Bureau Statistiek) were applied. Migration background was based on the woman’s parents’ country of birth or her own country of birth. Western is defined as originating from a country in Europe (excluding Turkey), North America and Oceania, or from Indonesia or Japan.

By comparing pregnancy complications in the COVID‐19 pandemic versus the prepandemic period, we observed similar prevalences of high blood pressure, gestational diabetes mellitus and placenta previa. However, in multiparous women, fetal growth restriction was suspected more often during the early COVID‐19 pandemic than in the prepandemic period (Table 2).

**TABLE 2:** Pregnancy and birth characteristics of the included VeCaS‐population of pregnant women in the prepandemic (2019, n = 2963) and COVID‐19 pandemic period (2020, n = 2950), total n = 5913

	2019			2020			Statistical differences between 2019–2020		
	Total	Primiparous	Multiparous	Total	Primiparous	Multiparous	Total	Primiparous	Multiparous
	n = 2963	n = 1317	n = 1646	n = 2950	n = 1319	n = 1631			
	100%	44%	56%	100%	45%	55%			
	n (%)	n (%)	n (%)	n (%)	n (%)	n (%)	*P*‐value^*^	*P*‐value^*^	*P*‐value^*^
Pregnancy complications									
High blood pressure	361 (12.2)	227 (17.2)	134 (8.1)	365 (12.4)	208 (15.8)	157 (9.6)	0.825	0.310	0.135
Diabetes mellitus	115 (3.6)	378 (2.9)	77 (4.7)	119 (4.0)	46 (3.5)	73 (4.5)	0.763	0.379	0.782
Suspected fetal growth restriction	111 (3.7)	62 (4.7)	49 (3.0)	133 (4.5)	62 (4.7)	71 (4.4)	0.141	0.993	**0.036**
Placenta previa	19 (0.6)	7 (0.5)	12 (0.7)	24 (0.8)	11 (0.8	13 (0.8)	0.436	0.346	0.823
Level of care at start delivery									
Primary care	1685 (56.9)	719 (54.6)	966 (58.7)	1678 (56.9)	726 (55.0)	952 (58.4)	0.992	0.817	0.853
Secondary care	1278 (43.1)	598 (45.4)	680 (41.3)	1272 (43.1)	593 (45.0)	679 (41.6)			
Onset of labor									
Spontaneous	2191 (75.0)	967 (74.9)	1224 (75.1)	2092 (72.4)	936 (73.1)	1156 (71.8)	0.068	0.513	0.103
Induction of labor ^a^	561 (19.2)	270 (20.9)	291 (17.9)	620 (21.4)	292 (22.8)	328 (20.4)			
Planned cesarean section	168 (5.8)	54 (4.2)	114 (7.0)	178 (6.2)	53 (4.1)	125 (7.8)			
Missing	43	26	17	60	38	22			
Augmentation of labor									
No	2546 (87.2)	1060 (82.0)	1486 (91.3)	2523 (86.9)	1037 (80.1)	1486 (92.3)	0.698	0.202	0.290
Yes	374 (12.8)	232 (18.0)	142 (8.7)	382 (13.1)	258 (19.9)	124 (7.7)			
Missing	43	25	18	45	24	21			
Pain medication^b^									
None	2064 (76.2)	799 (65.4)	1265 (85.1)	2025 (73.6)	768 (61.1)	1257 (84.0)	**0.025**	**0.015**	0.703
Epidural	408 (15.1)	294 (24.1)	114 (7.7)	466 (16.9)	354 (28.2)	112 (7.5)			
Remifentanil	190 (7.0)	96 (7.9)	94 (6.3)	231 (8.4)	117 (09.3)	114 (7.6)			
Pethidine	37 (1.4)	26 (2.1)	11 (0.7)	23 (0.8)	13 (1.0)	10 (0.7)			
Gas and air	9 (0.3)	7 (0.5)	2 (0.1)	8 (0.3)	5 (0.4)	3 (0.2)			
Missing	87	41	46	19	9	10			
Preferred place of birth									
Home birth	641 (22.8)	253 (20.2)	388 (24.9)	718 (26.0)	304 (24.8)	414 (27.1)	**0.017**	**0.044**	0.340
Midwife‐led hospital birth	1737 (61.7)	915 (73.1)	822 (52.7)	1549 (57.8)	836 (68.1)	758 (49.5)			
Obstetric unit	369 (13.1)	53 (4.2)	316 (20.2)	374 (13.6)	55 (4.5)	319 (20.8)			
Not decided	66 (2.3)	31 (2.5)	35 (2.2)	72 (2.6)	33 (2.7)	39 (2.5)			
Missing	150	65	85	192	91	101			
Actual place of birth									
Home birth	580 (20.1)	151 (11.9)	429 (26.5)	684 (23.8)	188 (14.8)	496 (31.0)	**<0.001**	0.076	**<0.001**
Midwife‐led hospital birth	543 (18.8)	165 (13.0)	378 (23.4)	417 (14.5)	149 (11.7)	268 (16.7)			
Obstetric unit	1768 (61.2)	957 (75.2)	811 (50.1)	1770 (61.7)	933 (73.5)	837 (52.3)			
Missing	72	44	28	79	49	30			
Mode of birth									
Spontaneous vaginal birth	2320 (80.1)	903 (71.0)	1417 (87.3)	2325 (81.1)	920 (72.6)	1405 (87.8)	0.385	0.626	0.279
Assisted vaginal birth ^c^	212 (7.3)	185 (14.6)	27 (1.7)	180 (6.3)	162 (12.8)	18 (1.1)			
Planned cesarean section	168 (5.8)	54 (4.2)	114 (7.0)	178 (6.2)	53 (4.2)	125 (7.8)			
Emergency cesarean section	195 (6.7)	129 (10.1)	66 (4.1)	185 (6.5)	133 (10.5)	52 (3.3)			
Missing	68	46	22	82	51	31			

^a^ Induction of labor comprises the following: amniotomy, oxytocin, pharmacological priming (misoprostol or PgE2), and mechanical priming with balloon catheter.

^b^ Pain medication during first stage of labor, calculated for vaginal births and emergency cesarean sections. Planned cesarean sections excluded.

^c^ Mode of birth: Assisted vaginal birth includes a vacuum or forceps extraction.

^*^ Values in bold indicate statistically significant results.

More women preferred to give birth at home during the COVID‐19 pandemic than in the prior year. For nulliparous women (24.8% vs 20.2%; *P* = 0.044), this difference was statistically significant (compared with multiparous women [27.1% vs 24.9%; *P* = 0.340]). More women also completed home births during COVID‐19 (23.8% vs 20.1%; *P* < 0.001). This increase was statistically significant for multiparous women (31.0% vs 26.5%; *P* < 0.001), but not for nulliparous women (14.8% vs 11.9%; *P* = 0.076).

Although no statistical differences were observed for mode of birth, the rate of vaginal births increased. Compared with the prepandemic period, a small decrease in emergency cesareans and a small increase in planned cesarean births were observed during the COVID‐19 pandemic. During the pandemic, fewer women gave birth without pain medication (73.6% vs 76.2%, *P* = 0.025). This difference was statistically significant for nulliparous women (61.1% vs 65.4%; *P* = 0.015), but not for multiparous women (84.0% vs 85.1%, 156 *P* = 0.703).

We observed a lower incidence of episiotomy in 2020 compared to 2019, which was statistically significant for multiparous women at 5.0% versus 6.9% respectively (*P* = 0.032). However, there were no changes in the COVID‐19 pandemic versus the prepandemic period in blood loss after birth or in 3rd/4th degree tears both among nulliparous and among multiparous women. These incidences were similar to those in the total population (Table 3).

**TABLE 3:** Maternal and infant health outcomes of the included VeCaS‐population of women in the COVID‐19 pandemic period (2020) versus the prepandemic period (2019), total n = 5913

	2019			2020			Statistical differences between 2019–2020		
	Total	Primiparous	Multiparous	Total	Primiparous	Multiparous	Total	Primiparous	Multiparous
	n = 2963	n = 1317	n = 1646	n = 2950	n = 1319	n = 1631			
	100%	44%	56%	100%	45%	55%			
	n (%)	n (%)	n (%)	n (%)	n (%)	n (%)	*P*‐value^*^	*P*‐value^*^	*P*‐value^*^
Maternal health outcomes									
Episiotomy^a^									
No	2101 (82.1)	748 (67.6)	1353 (93.1)	2149 (84.4)	780 (70.5)	1369 (95.0)	**0.03**	0.141	**0.032**
Yes	458 (17.9)	358 (32.4)	100 (6.9)	398 (15.6)	326 (29.5)	72 (5.0)			
3rd/4th degree tear^a^									
No	2489 (97.2)	1064 (96.1)	1425 (98.1)	2464 (96.7)	1047 (94.7)	1417 (98.3)	0.311	0.141	0.596
Yes	71 (2.8)	43 (3.9)	28 (1.9)	83 (3.3)	59 (5.3)	24 (1.7)			
Blood loss after delivery in ml									
≤499	2167 (76.5)	894 (71.7)	1273 (80.4)	2155 (77.0)	877 (71.0)	1278 (81.8)	0.816	0.167	0.443
500–999	574 (20.3)	308 (24.7)	266 (16.8)	549 (19.6)	299 (24.2)	250 (16.0)			
1000–1999	61 (2.2)	29 (2.3)	32 (2.0)	68 (2.4)	47 (3.8)	21 (1.3)			
≥2000	29 (1.0)	16 (1.3)	13 (0.8)	26 (0.9)	12 (1.0)	14 (0.9)			
Missing	132	70	62	152	84	68			
Infant health outcomes									
Gestation at birth									
Preterm (<37 weeks)	137 (4.6)	84 (6.4)	53 (3.2)	124 (4.2)	88 (6.7)	36 (2.2)			
Extremely preterm (< 28 weeks)	5 (0.2)	4 (0.3)	1 (0.1)	3 (0.1)	2 (0.2)	1 (0.1)	0.431	0.760	0.075
Very preterm (28–31 + 6)	12 (0.4)	5 (0.4)	7 (0.4)	19 (0.6)	14 (1.1)	5 (0.3)			
Moderate to late preterm (32–36 + 6)	120 (4.0)	75 (5.7)	45 (2.7)	102 (3.5)	72 (5.5)	30 (1.8)			
Term (≥37 weeks)	2826 (95.4)	1233 (93.6)	1593 (96.8)	2826 (95.8)	1231 (93.3)	1595 (97.8)			
Term (37–41 + 6)	2779 (93.8)	1206 (91.6)	1573 (95.6)	2787 (94.5)	1205 (91.4)	1582 (97.0)			
Post term (≥42 weeks)	47 (1.6)	27 (2.1)	20 (1.2)	39 (1.3)	26 (2.0)	13 (0.8)			
Gender									
Female	1392 (47.3)	621 (47.7)	771 (47.0)	1396 (47.6)	622 (47.8)	774 (47.5)	0.781	0.954	0.749
Male	1552 (52.7)	682 (52.3)	870 (53.0)	1534 (52.4)	680 (52.2)	854 (52.5)			
Missing	19	14	5	20	17	3			
Birthweight (percentiles)^b^									
<p10	284 (9.8)	187 (14.7)	97 (6.0)	268 (9.3)	175 (13.8)	93 (5.8)	0.517	0.538	0.792
≥p10	2611 (90.2)	1088 (85.3)	1523 (94.0)	2611 (90.7)	1092 (86.2)	1519 (94.2)			
Missing	68	42	26	71	52	19			
Apgar score after 5 min									
0–3	50 (1.7)	31 (2.4)	19 (1.2)	38 (1.3)	16 (1.3)	22 (1.4)	0.437	0.081	0.565
4–6	101 (3.5)	55 (4.3)	46 (2.8)	97 (3.4)	60 (4.7)	37 (2.3)			
7–10	2753 (94.8)	1191 (93.3)	1562 (96.0)	2750 (95.3)	1200 (94.0)	1550 (96.3)			
Missing	59	40	19	65	43	22			
Perinatal mortality (≤28 days postpartum)									
No	2924 (99.7)	1291 (99.4)	1633 (99.9)	2917 (99.7)	1297 (99.7)	1620 (99.8)	0.641	0.246	0.409
Yes	10 (0.3)	8 (0.6)	2 (0.1)	8 (0.3)	4 (0.3)	4 (0.2)			
Missing	29	18	11	25	18	7			

^a^ Birthweight in percentiles according to the perined birthweight charts.

^b^ The maternal outcomes episiotomy and 3rd and 4th degree tears are calculated for vaginal births only (total population n = 2560 (2019) and n = 2547 (2020). * Values in bold indicate statistically significant results

^*^ Values in bold indicate statistically significant results.

No statistically significant differences were found in neonatal health outcomes in the COVID‐19 pandemic versus the prepandemic period, although we noticed a small, nonsignificant decrease in the incidence of preterm births (4.2% vs 4.6%).

## 4 DISCUSSION

In this study, we examined whether the course of pregnancy and birth among low‐risk women who started their care in primary midwifery care, and the accompanying maternal and neonatal health outcomes differed in the early COVID‐19 period (2020) compared to the prepandemic period (2019). We found that during the pandemic, both nulli‐ and multiparous women more often preferred to give birth at home and that there were more home births. In addition, there was a lower incidence of episiotomy. During the COVID‐19 pandemic, women more often used pain medication.

Before this study, the possible effect of the pandemic on preferred and actual place of birth in the Netherlands was unknown. Since the Netherlands has a maternity care system with a relatively high rate of home births, our findings may not be generalizable to other high‐income countries. However, in various other countries too, increasingly women have a choice in place of birth.^17^, ^18^

Our findings align with other research showing that the interest in home birth increased during the COVID‐19 pandemic.^7^, ^19^ Due to the pandemic, health care services changed rapidly and attempted to balance between reducing the risk of transmission and maintaining high‐quality care. Several studies showed that pregnant women were concerned about entering the hospital and that the pandemic affected their intended place of birth.^19^ Nelson et al^20^, ^21^ described several possible reasons for this: fear of acquiring an infection from a hospital visit, fear of being unsupported as a result of restrictive policies in relation to birth partners and visitors, and fear about access to pain relief or to water birth. Given the measures taken in Dutch maternity care during the pandemic, these reasons could also apply to the Netherlands. A survey conducted in the Netherlands among maternity caregivers showed that respondents felt that women had more confidence in giving birth at home.^22^ Although restrictions and fear are not good motives for choosing home birth, over the past decades, unfounded fear of home birth has led to increasing numbers of women choosing a hospital birth.^23^ Women weigh the advantages and disadvantages of different places of birth; due to COVID‐19, this balance may have shifted more often toward home birth.^22^, ^24^

This may be viewed as a positive development. The International Confederation of Midwives (ICM) states that in countries where the health systems can support home birth with care from qualified midwives with appropriate emergency equipment, healthy women experiencing a normal pregnancy may be safer birthing at home or in a primary maternity unit/birth center than in a hospital where there may be many patients (even nonmaternity patients) with COVID‐19.^25^

Conversely, choice in place of birth, as with choices in other aspects of care, should be a free choice for pregnant women and not motivated by a restrictive maternity care system or fear of getting infected in a hospital. This is important since women’s choices and sense of control and autonomy impact birthing experiences; it is well known that in addition to physical maternal and neonatal health outcomes, women’s birth experiences are important indicators of high‐quality care.^26^

As stated by the WHO, all pregnant women and their newborns, including those with confirmed or suspected COVID‐19 infections, have the right to high‐quality care before, during and after childbirth, including mental health care.^27^, ^28^

The lower incidence of episiotomy may be related to the decrease in the national incidence of episiotomy over the last decade.^29^ However, the lower incidence of episiotomy could also be related to the increased number of home births, since previous research on Dutch childbirth intervention rates showed that the episiotomy rate in primary midwife‐led care was lower than the episiotomy rate in secondary obstetrician‐led care.^30^

The higher incidence of the use of pharmacological pain relief during labor is in accordance with the Dutch national data showing that in 2019, in 21.2% of all births (including cesarean sections) epidural analgesia was applied. In 2020, this percentage had increased to 22.4%.^29^

Although fetal growth restriction was suspected more often among multiparous women, the rate of babies that were small for gestational age was similar before and during the COVID‐19 pandemic. The increased levels of anxiety among women during the pandemic may have resulted in more worries about their baby’s growth and requests for an extra ultrasound. Ultrasounds during pregnancy are known to have a low specificity, and therefore, babies may be suspected incorrectly of being small for gestational age.^31^

Another Dutch study showed that the initial implementation of COVID‐19 measures was associated with a substantial reduction in the incidence of preterm births in the first month during the initial wave of the COVID‐19 pandemic.^32^ In our study, we did not observe a statistically significant difference of (very) preterm birth when comparing the prepandemic and COVID‐19 pandemic periods. Both studies included singleton births from 24 weeks of gestation onwards; however, our study included women who received care from primary midwife, whereas Been et al^32^ included women who received midwife‐led care or obstetrician‐led care. In general, women in obstetrician‐led care are high‐risk women who might be more likely to give birth preterm.

### 4.1 Strengths and limitations

This is the first study to investigate the influence of the COVID‐19 pandemic on pregnancy and birth outcomes among low‐risk pregnant women, starting their antenatal care in midwife‐led care. Detailed data from the unique VeCaS data set were used from 39 midwifery practices throughout the Netherlands. Using registration data for research might be seen as a limitation, since data were not specifically collected to answer the research question. However, our study is purely descriptive and statistics are used accordingly, since our goal was to describe changes in pregnancy and birth, and implications for women’s choice of and actual place of birth related to the pandemic. In our study population, we showed that the pandemic affected women’s childbirth care, suggesting that women’s choices for place of birth are influenced by their perception of risk and autonomy, as is known from other studies.^33^ This emphasizes once more the need for a thorough exploration of perceptions when discussing place of birth among healthy, low‐risk women. A maternity care system in which home births are well integrated may be more resilient to extreme health care challenges such as pandemics.

Further exploration of the effect on other variables, such as the impact of mental health factors during the pandemic, is recommended. There is a need for qualitative research to explore women’s experiences of pregnancy and birth during this pandemic.

### 4.2 Conclusions

During the COVID‐19 pandemic, there was a higher rate of planned and actual home birth, and suspected growth restriction, and a lower rate of episiotomy among low‐risk women in the Netherlands.

## ETHICAL APPROVAL

Ethical approval for the VeCaS database was obtained from the regional Medical Research Ethics Committee of Academic Hospital Maastricht and the University of Maastricht (METC azM/UM, nr 09–4‐061). Because of its noninvasive nature, this type of research does not require further ethical approval in the Netherlands.

## Data Availability

The data that support the findings of this study are available on request from the corresponding author. The data are not publicly available due to privacy or ethical restrictions.
